# Accuracy of collagen fibre estimation under noise using directional MR imaging

**DOI:** 10.1016/j.compmedimag.2020.101796

**Published:** 2020-12

**Authors:** Djordje Brujic, Karyn E. Chappell, Mihailo Ristic

**Affiliations:** a Mechanical Engineering Department, Imperial College London, London, UK; b MSK Lab, Department of Surgery and Cancer, Imperial College London, UK

**Keywords:** Collagen, Magic angle, Magnetic resonance imaging, Tractography

## Abstract

In tissues containing significant amounts of organised collagen, such as tendons, ligaments, menisci and articular cartilage, MR imaging exhibits a strong signal intensity variation caused by the angle between the collagen fibres and the magnetic field. By obtaining scans at different field orientations it is possible to determine the unknown fibre orientations and to deduce the underlying tissue microstructure. Our previous work demonstrated how this method can detect ligament injuries and maturity-related changes in collagen fibre structures. Practical application in human diagnostics will demand minimisation of scanning time and likely use of open low-field scanners that can allow re-orienting of the main field. This paper analyses the performance of collage fibre estimation for various image SNR values, and in relation to key parameters including number of scanning directions and parameters of the reconstruction algorithm. The analysis involved Monte Carlo simulation studies which provided benchmark performance measures, and studies using MR images of caprine knee samples with increasing levels of synthetic added noise. Tractography plots in the form of streamlines were performed, and an Alignment Index (AI) was employed as a measure of the detected orientation distribution. The results are highly encouraging, showing high accuracy and robustness even for low image SNR values.

## Highlights

• Accurate and efficient estimation of collagen fibre directions by exploiting the Magic Angle effect.
• Scan optimisation significantly reduces the required number of scans at different field orientations and the overall scanning time.
• High accuracy of computation of fibre directions of less than one degree, with significantly improved computing speed.
• Highly robust performance in the presence of image noise, indicating compatibility with low field MRI in future applications.

## Graphical abstract

**Figure:**
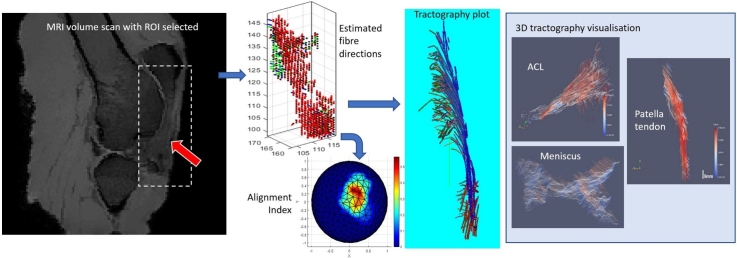

## 1 Introduction

Conventional MRI cannot accurately diagnose tissues containing collagen as its main component, such as ligaments, tendons and menisci. Consequently, invasive procedures such as arthroscopy remain as the diagnostic gold standard, even though they are normally undertaken only as therapy. Conditions such as partial ligament tears are particularly difficult to diagnose using traditional MR imaging, which provides no functional assessment of the remaining portion (Devitt et al., 2017; Temponi et al., 2015). The knee is the most affected joint in injuries, with annual incidence of 61 meniscal tears and 30 cruciate ligament injuries per 100,000 population (Kurtz et al., 2007). Knee osteoarthritis affects 4.7 Million people in the UK alone, with over 90,000 knee replacements per year (Willis-Owen et al., 2009) and there is a trend for younger patients to require replacement due to wear after injury. In the case of knee replacement surgery, the choice between total knee replacement, and the less radical unicompartmental replacement, or cruciate sparing replacement, also critically depends on the accuracy of assessing the state of the ligaments (Mancuso et al., 2016).

In ligaments and tendons, collagen forms a hierarchical structure of fibrils, fibres and fascicles, and fibre structures can also be identified in other tissues. At physiological hydration levels, 89 % of the water present in the tissue is bound to collagen (Fullerton and Rahal, 2007) in chain-like structures, while the remainder is the water in gaps. In collagen bound water the unaveraged dipolar interactions of proton nuclear spins are the dominant signal decay mechanism, causing a very short transverse relaxation times $T_{2}$ and $T_{2}^{\text{*}}$, and a generally low MR image signal intensity. It is well known (Berendsen, 1962; Bydder et al., 2007; Henkelman et al., 1994) that the dipolar interactions are modulated by the term $\left(3{cos}^{2}\theta -1\right)$, where θ is the angle between the proton-proton direction and the main magnetic field $B_{0}$, and this will diminish for θ=54.7°, commonly known as the magic angle (MA). As a result, the measured $T_{2}$ may be extended from about 1−2 ms for θ=0 to perhaps more than 20 ms when θ is the magic angle. For imaging sequences with relatively short echo times, typically if TE < 37 ms, this will result in marked changes in the observed image intensity, from very dark (almost no signal) to very bright. In musculoskeletal MR imaging this phenomenon is usually considered to be a source of artefacts, because healthy ligaments would generally appear black, while regions of high intensity may be a sign of a disease or injury, or an artefact due to MA.

Previous work (Seidel et al., 2013; Szeverenyi and Bydder, 2011) has shown that by measuring the variation of signal intensity with θ it is possible to deduce information about the tissue microstructure, which cannot be easily obtained by other non-invasive means. The recorded image intensity of each voxel obtained at various angles θ can be used to estimate the dominant collagen fibre orientation. The resulting data may be used to generate 3D tractography plots, which are visually similar to the results obtained using Diffusion Tensor Imaging (DTI) but involve an entirely different contrast mechanism. In our recent work (Chappell et al., 2019) we proposed an improved method for selecting optimised scan directions leading to a reduced number of scans at different field angles θ, and an improved method to calculate collagen fibre orientations leading to a significantly reduced number of computations and improved accuracy. This method was successfully employed to detect previously unknown ligament injury, and maturity-related changes in the collagen structure of knee specimens.

The work presented in this paper was conducted to investigate the performance of this method, and particularly its dependence on the image signal-to-noise ratio (SNR). The main motivation is that the potential future applications in humans will depend on the use of open scanners, since in the confines of conventional closed bore scanners it is mostly impossible to change the angle of the main field to the body. Low-field open scanners based on permanent magnets have found wide adoption for musculoskeletal imaging, while novel permanent magnet configurations have also been proposed as a way to fully exploit field-related anisotropies (McGinley et al., 2018). Since the estimation of collagen fibre directions using directional imaging involves the analysis of image intensity variation in each voxel, a key question is how sensitive this approach is to image noise. In the attempt to provide adequate answers, we have conducted Monte Carlo simulation studies in combination with experimental imaging using animal samples and various levels of artificially added noise. In Section II, our method for Magic Angle Directional Imaging (MADI) method is summarised, together with the definition of the Alignment Index as an adopted metric relating to the degree of fibre anisotropy. Subsequent sections present the details of our simulation studies, experimental imaging and results.

## 2 Methods

The overall imaging and image processing procedure for Magic Angle Directional Imaging (MADI) is summarized in the following steps, while further details are provided in subsequent subsections.

1. MR Imaging

Volume images of the subject are obtained at  N equally distributed orientations of the main field $B_{0}$ relative to the subject.

2.Registration of volume images

This is performed to establish voxel correspondences, such that image intensity variation can be analysed, and the rotation matrix obtained as part of the transformation is used for the subsequent calculation of fibre directions.

3 Segmentation

The region of interest (ROI) is interactively selected by the user. Voxels within ROI exhibiting intensity variation above a predefined threshold are segmented for further processing.

4 Estimation of fibre directions

For each voxel in the set, the measured intensity values are correlated with those predicted using theoretical relationships, and the collagen fibre direction vector is estimated using cost function minimization.

5 Visualization and metrics

The results may be visualised using suitable 3D vector field plots or used to construct 3D tractography plots using streamlines. In addition, distribution of collagen fibre orientations and metrics such as the Alignment Index (AI) may be calculated and visualized.

All software was developed in Matlab (Mathworks, Inc.), except for tractography visualization which was performed using ParaView (Sandia National Laboratory).

### 2.1 Scanning orientations

The number of required scans at different orientations of $B_{0}$ to the subject is of great practical importance because it directly determines the overall scanning time. Prior work involved relatively large numbers of scans and the selected orientations were not optimised, as they were mainly chosen for experimental convenience. Szeverenyi et al. (Szeverenyi and Bydder, 2011) performed scanning at 15 orientations, which were confined to 3 orthogonal planes. Seidel et al. (Seidel et al., 2013) used 35 orientations, which were confined to 2 orthogonal planes. The approach that we adopted is as follows.

**Fig. 1:**
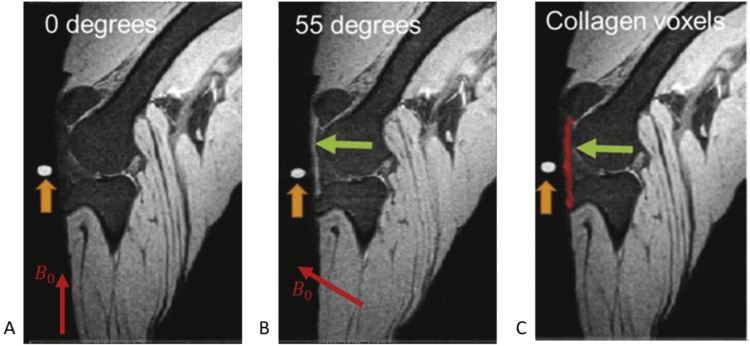
9 equidistant orientations represented by points on a hemisphere. $\left[0,0,1\right],\;\left[0.42,0.42,0.8\right],\;\left[-0.42,0.42,0.8\right],\;\left[-0.42,-0.42,0.8\right],\;\left[0.42,-0.42,0.8\right],\;\left[0.92,0,0.4\right],\;\left[0,\;0.92,0.4\right],\;[-0.92,0,0.4],\;[0,-0.92,0.4]$

It is apparent that if a number of scanning directions were confined in one plane, they would produce similar intensities for all collagen fibre direction being approximately normal to that plane, so the ability to detect the angular anisotropy of those fibres would be severely limited. We can conclude that for optimal results no three scanning directions should be chosen to lie in the same plane. Based on this we postulated that the selected orientations of $B_{0}$ should be equidistant in 3D in order to provide a better sampling of the intensity variation function. Equidistant orientations are those that would maximise the minimum angle between them, and they may be found by using a method to partition a unit sphere into regions of equal area. The directions belonging to one hemisphere can then be selected. We have used the method for partitioning the sphere proposed in (Leopardi, 2006) and Fig. 1 gives an example for the case of 9 equidistant directions.

### 2.2 Fiducial localization and registration

Registration of volume images was performed using three passive markers, consisting of 10 mm diameter water filled balls, which were fixed with the sample. Localization of the markers in the images was performed using the template matching technique, which relies on their spherical shape. Similarly to (de Oliveira et al., 2008) the Fourier Filtering Method (Parker, 2010) is employed, however, here it is applied to a volume instead of the 2D images. If M is the Fourier transforms of the acquired volume image and J is that of a template sphere of known radius, then:

(1) M=J×Δ+N

where N is the noise and Δ is the Fourier transform of the delta functions that identifies the position of the sphere. If a Wiener filter is used to reduce the noise produced by other objects, then N may be neglected in (1) and the sphere position (x, y, z) can be calculated using the inverse Fourier transform:

(2) $$\delta (x,\;y,z)={FFT}^{-1}(M/J)$$

The division of the Fourier transformed images M and J is computed by dividing the magnitudes and subtracting the phase values. The voxels in the region with the highest intensity correspond to the template sphere. Sub-voxel accuracy may then be achieved by calculating that region’s centre of mass weighted by the intensity. Previous work (Franco et al., 2016) indicated that the typical accuracy of the localization method is better than 0.3 voxels.

If the markers are arranged in a known pattern such that they are not equidistant from each other, then the correspondence between identified marker positions in different volume images can be automatically established (Galassi et al., 2015). Registration is then performed as a rigid body transformation that minimizes the sum of the squared distances between the corresponding marker points (Arun et al., 1987). In this way, with one of the N acquired image volumes taken as a reference, the other N-1 volumes acquired at different orientations are registered to it, and the corresponding rigid body transformation matrices are recorded. In the final step, nearest neighbour method is used to establish voxel-voxel correspondences between all acquired volumes.

In the presence of any image distortions, which are often caused by the non-linearity of the gradients and non-uniformity of the main field, it was found that the results of the rigid body registration were improved by performing an additional fine alignment manually. This involved only translations of typically less than one voxel (approximate voxel size $1{mm}^{3}$), but it can significantly improve the accuracy in the selected region of interest. Interactive graphical tools were developed for this purpose, where the first slice is overlapped with each of the other scans in turn. We browse through slices until we find ROI, then we manually translate the second slice left, right, up and down until we find the best overlap.

### 2.3 Segmentation

Having found corresponding voxels, a 3D map of standard deviation of intensity is computed and visualised. A region of interest (ROI) may be interactively selected as a rectangular block of voxels. Voxels within ROI with standard deviation of intensity above a prescribed threshold are assumed to contain oriented collagen and are segmented for subsequent processing.

### 2.4 Computation of fibre orientations

The segmented voxels each have a vector of *N* intensities IM that correspond to N scanning directions. According to the analysis presented in (Szeverenyi and Bydder, 2011) the variation of the image intensity with the angle θ between the collagen fibres and the the field $B_{0}$ may be adequately represented using the relationship

(3) $$I=A\;exp\left({-B\left(3{\text{cos}}^{2}\theta -1\right)}^{2}\right)$$

The constants A and B are chosen such that the range of values of I matches the observed range of intensities in the segmented voxels. The value of A corresponds to the maximum observed intensity value, while B corresponds to the difference between the observed the maximum and the minimum intensities. This equation can then be used to compute a set of N theoretical intensity values IC.

In order to estimate the unknown orientation x of the fibres, we construct the following cost function for each voxel:

(4) $$f\left(x\right)=\;\sum_{k=1}^{N}{\left({IM}_{k}-\;{IC}_{k}(x)\right)}^{2}$$

where ${IM}_{k}$ represents the measured intensity of the voxel scanned in the k-th direction, ${IC}_{k}(x)$ represents the computed theoretical intensity for this voxel, scanned in the k-th direction, that would be obtained if the fibre direction is x. The direction $x=\left(\alpha ,\beta \right)$ is defined in spherical coordinate system and the minimisation of $f\left(x\right)$ involves 2 dimensions. The cost function (4) was minimised using the Nelder-Mead simplex algorithm (Lagarias et al., 1998), which does not require computation of derivatives. The algorithm starts by first making a simplex of 3 points around an initial guess $x_{0}$.

The cost function is highly non-linear and involves multiple minima, particularly in the presence of noise, so it is necessary to provide a sufficiently accurate initial guess to achieve convergence. In order to find the initial guess, we compute *M* test directions regularly distributed on a hemisphere (Leopardi, 2006). For each test direction we compute *N* theoretical intensities using Eq. (3), i.e. one for each actual scanning direction as determined in the registrations step. For each segmented voxel correlations between its set of intensities and those found for each test direction are calculated, and the direction giving the largest correlation value was assumed to be the initial guess $x_{0}$ for that voxel. The search for initial guess is similar to the method suggested by (Szeverenyi and Bydder, 2011). The main difference is that the orientation that they take as a final result is only an initial guess for our minimisation (4), and we also compute the correlation coefficient by its formal definition.

### 2.5 Alignment index (AI)

We adopted the Alignment Index (AI) as a suitable metric describing the degree of anisotropy in the computed vector field, which may be calculated for any specific orientation. For a chosen orientation, we consider the fraction of the calculated orientations that are within a 20° solid angle of a cone centred at that orientation, and a fraction of the same number of random orientations that are within the same solid angle. The 20° solid angle was chosen somewhat arbitrarily, following some experimentation with different angle values applied to datasets collected in our experiments.

AI may be calculated for any chosen orientation as follows. If $n_{Total}$ is the total number of fibre orientations considered (i.e. the number of segmented voxels),and $n_{M}$ is the number of those within a 20° solid angle from the specified orientation, we generate a set of $n_{Total}$ random orientations and find the number $n_{Rnd}$ of those within the same 2 0° solid angle. AI is then calculated using the following equations:

$$\;\text{i}\text{f}\;n_{M}\geq n_{Rnd\;then\;AI=\frac{\left(n_{M}-n_{Rnd}\right)}{\left(n_{Total}-n_{Rnd}\right)}}$$

(5) $$if\;n_{M}<n_{Rnd}\;then\;AI\;=\;0$$

Note that AI is normalised in such a way that AI = 0 indicates a fully isotropic vector set, while increasing AI values indicate increasingly aligned vector sets. AI = 1 corresponds to all vectors being orientated within the 20° solid angle from the selected direction.

**Fig. 7:**
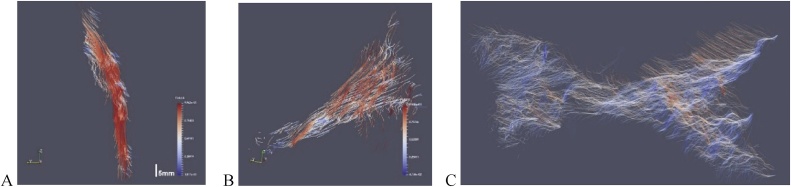
Representative results obtained for different SNR values, showing the images, tractography plots for the patella tendon and plots of *Alignment Index* distribution for SNR = 80, 20 and 5. The dashed box in the images indicates the selected region of interest and the arrows indicate the patella tendon.

In order to visualise the orientation distribution in a dataset we calculate AI for 1000 equidistant points on a hemisphere, from which the direction and the value of maximum AI can also be found. The equidistant points were calculated using the algorithm in (Semechko, 2018), which performs for uniform sampling of a sphere. 3D plots of the alignment index can be seen for the results presented in Fig. 7, where the magnitude of AI is coded in colour.

### 2.6 Simulation studies

Monte Carlo simulations were conducted in order to characterise the adopted methods in relation to imaging noise and to provide a benchmark performance against which experimental results can be compared. In particular we investigated the following:

• Robustness in terms of the number of scans and signal to noise ratio
• Accuracy of estimating fibre orientations
• Computational efficiency in relation to the number of template directions M.

Cases involving sets of between 7 and 11 scanning orientations were considered. Simulations in each case involved 10^5^ trials in which a single voxel was considered, and fibre orientation was randomly defined. Angle dependent signal intensity was determined using Eq. (3) with constants A = 250 and B = 0.4 providing the best fit to the range of intensities observed in our MR imaging experiments. Randomly generated Gaussian noise corresponding to various SNR values was added to these theoretical values. SNR was calculated as mean signal intensity divided by StDev of noise. In this context the mean is the average intensity obtained for the N scanning directions.

### 2.7 MR image data

Caprine legs were purchased from a local meat supplier who could ensure that the goat had been slaughtered and then refrigerated at 4 °C for no more than five days. The caprine leg was shortened by the butcher using a saw and blade to 175 mm with the joint line centralised at around 87 mm. The ends of the leg that had been cut were cleaned thoroughly in running water to remove any metal debris from the blade and saw which could cause susceptibility artefacts on the MRI images. The knee was wrapped in polythene to prevent leakages and to maintain tissue hydration. To prevent the knee flexing and returning to its neutral position during the scan clear parcel tape was used to immobilise the leg in an extended position (clear polypropylene tape, 41 μm x48 mm x66 m). Tape was applied to the proximal end pulled tight along the anterior aspect and fixed at the distal portion. Additional clear tape was applied to stabilise the quadriceps, hamstring muscles and other soft tissues.

**Fig. 2:**
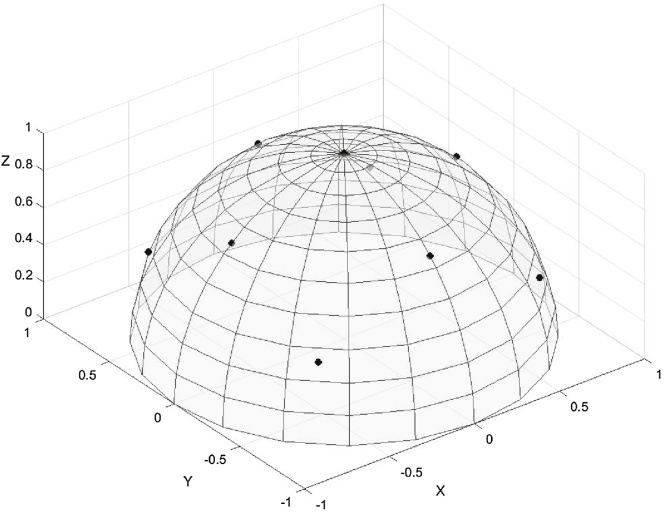
Goat knee sample mounted in the test sphere and placed within the 12 channel head coil in specially designed holder.

The sample (Fig. 2) was mounted inside a plastic test sphere, by embedding the distal and proximal ends in plasticine, which was in previous tests verified to have no MRI signal. This ensured that there was no movement of the sample within the sphere, which was set at different orientations relative to $B_{0}$ with the aid of suitable markings. Three water filled balls with a diameter of 10 mm (.43 Caliber Clear Paintballs 8000, Rap4 UK) were fixed to the surface of the sample at unequal distances to each other.

Imaging was performed with a 3 T scanner (Magnetom Verio, Siemens, Erlangen, Germany) with a 12-channel head coil as the signal receiver. Scans employed a 3D T1 FLASH (Fast Low Angle Shot) sequence with 1 mm isotropic voxels (TR 13 ms, TE 4.9 ms, FOV 256 mm).

The obtained MR images served as the image quality gold standard. Different levels of synthetic noise were subsequently added to these images in order to produce images with reduced SNR values, which were analysed using the DICODE method and compared.

## 3 Results

### 3.1 Number of field orientations

**Fig. 3:**
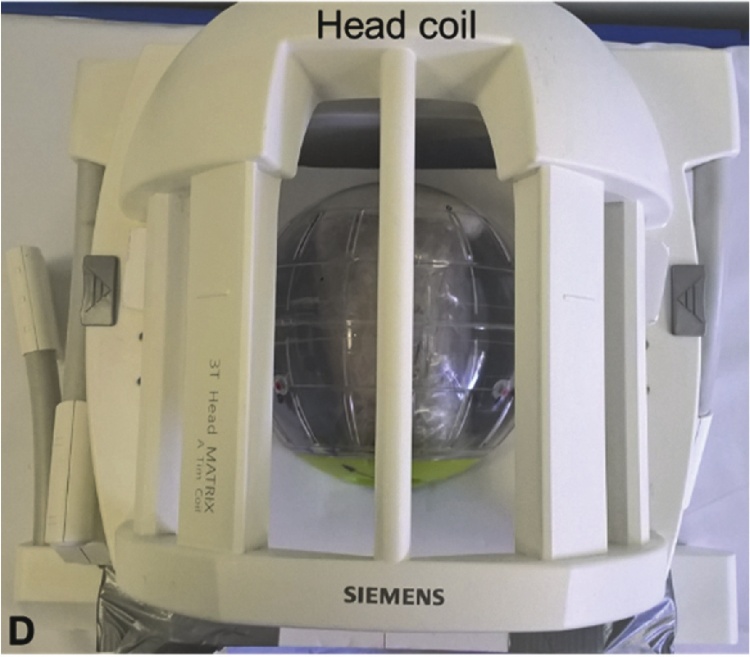
Monte Carlo simulation results showing percentage of failed calculations (error >10°) as a function of SNR, for different numbers of scan orientations equispaced in 3D.

We conducted Monte Carlo simulations in order to establish the required number of scanning orientations in the presence of image noise (105 simulation runs per configuration) involving simulated image noise SNR = 5, 10, 20, 30, 80 and N = 7, 9, 11 scanning orientations. In each case we recorded the number of simulations that failed to converge correctly, which was again judged by the error in calculating the orientation being >10°. The results are summarised in Fig.3, showing the percentage of failed calculations as a function of SNR, for different values of N.

The simulations show that even for SNR=10, using 7 scanning directions achieves 95.46 % of correct results, while using 9 scanning directions we achieve 98.75 % correct results. Using only 7 scanning directions we achieve 99.88 % success rate if SNR>20. Based on the above we have decided to use 9 scans in all our subsequent experiments.

### 3.2 Initial guess and robustness

**Fig. 4:**
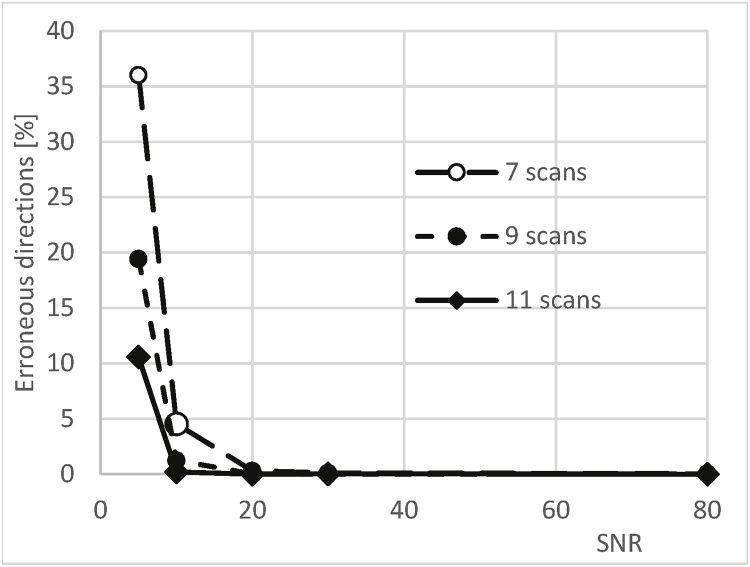
Percentage of 10^5^ Monte Carlo simulation runs that failed to reach global minimum (error>10°) vs. number of test directions used to find the initial guess, 7 scans with SNR = 30 and 9 scans with SNR = 10, 30.

Monte Carlo simulations were used to find the required number of test directions to be examined in order to provide a sufficiently accurate initial guess for minimization of Eq. (5). It was assumed that the minimisation did not reach the global minimum if the error in estimating fibre direction was greater than 10°. As the directions are computed from the intensities contaminated by noise, the observed error is mainly a result of the noise and to a much lesser extent due to an inherent inaccuracy of the iterative method. For different test sizes we employed 10,000 simulation runs for 7 scanning directions with SNR = 30, and for 9 scan directions with SNR = 10, 30. The results are summarised in Fig. 4 showing the percentage of runs that failed to convergence to global minimum. It can be seen that with 200 template directions in case of 9 scanning directions there will be less than 1% of erroneous results even for SNR = 10. Consequently, we adopted 250 test directions when 9 or more scanning directions were used, and 500 test directions when only 7scans were used.

### 3.3 Accuracy of estimation of fibre directions

**Fig. 5:**
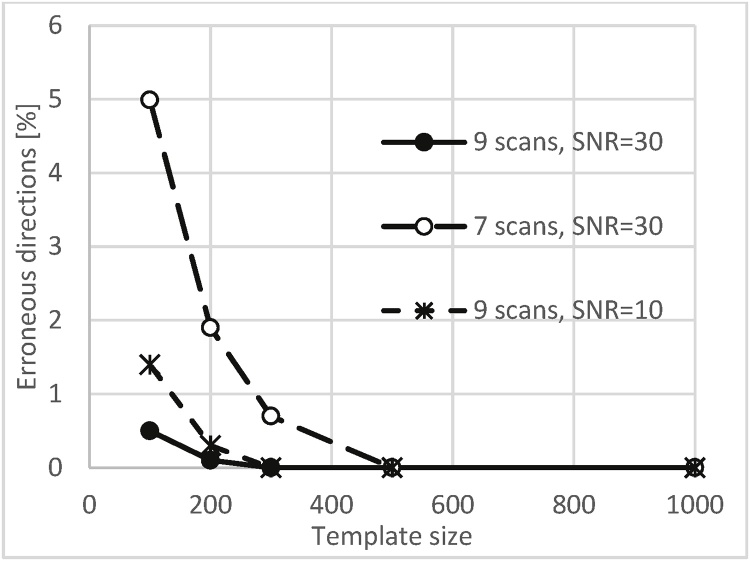
Mean (a) and standard deviation (b) of angular error using the proposed method with 7, 9 and 11 equidistant scan orientations. Results obtained using the method in (Szeverenyi and Bydder, 2011) are included for comparison.

Monte Carlo simulation involving $10^{5}$ tests was performed and Mean error and Standard deviation were examined as a function of SNR. Fig. 5 shows the results for 7, 9 and 11 equidistant scanning orientations, together with those obtained using the method in (Szeverenyi and Bydder, 2011), for comparison, showing that the improved method for fibre directions computations achieves much higher accuracy. Based on this, the number of scanning orientations in the imaging experiments was set to  N=9, considering the anticipated range of SNR values and the desired robustness of calculation.

### 3.4 Computational efficiency

It was found that on average 65 iterations of the simplex minimisation were required for a tolerance of 0.01°. The overall computational time was found to increase roughly linearly with the number of test directions used to determine the initial guess. The computing times per voxel for the calculation of fibre direction is presented in Table 1, where the computing times for the method in (Szeverenyi and Bydder, 2011) are included for comparison, though its accuracy is considerably lower, of the order of 2−3°.

**Table 1:** Computing times per voxel to calculate fibre directions for different numbers of test directions. The times for the previous method in (Szeverenyi and Bydder, 2011)are provided for comparison.

	*Previous Method*	Simplex minimisation					
Number of test dirs.	*900*	1000	750	500	250	200	100
Time (ms)	*90*	100	76	51	27	22	13

### 3.5 Sample tractography plots

**Fig. 6:**
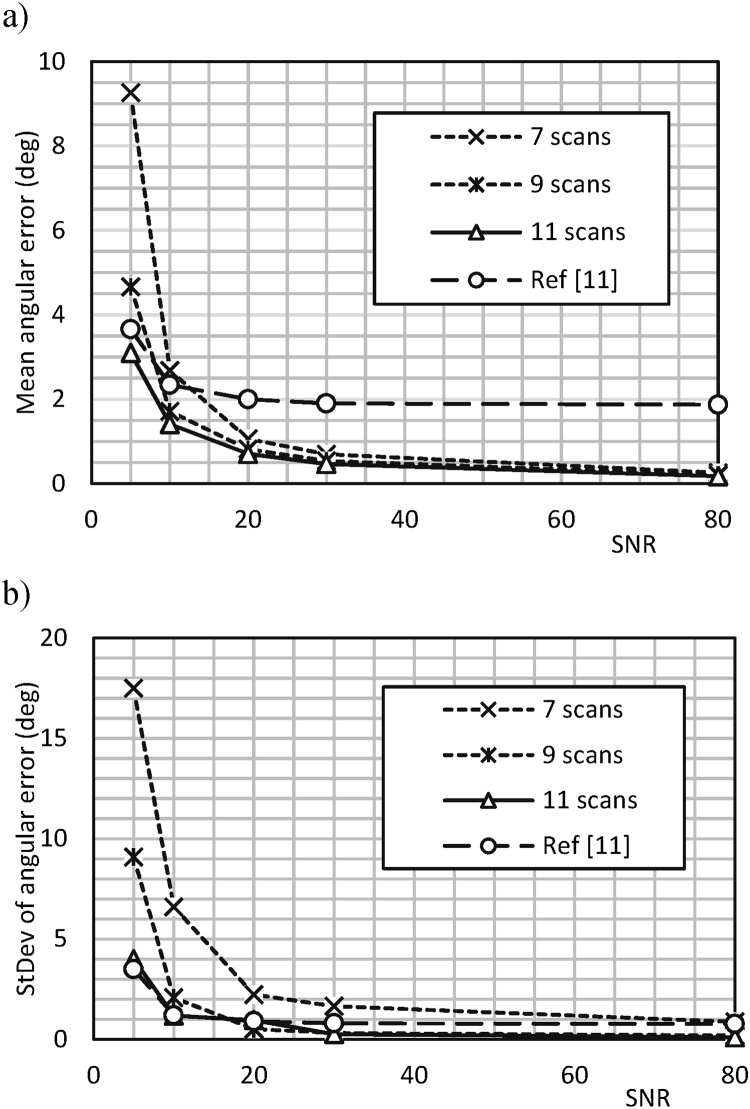
Example tractography results for different regions of interest: A: patella tendon, B: anterior cruciate ligament (ACL), C: the meniscus. The data was obtained from 9 vol scan orientations equally spaced in 3D.

Vector field data may be used to construct tractography plots in the form of streamlines, which originate from suitably selected seed points and are calculated using the direction vectors computed for each voxel in the segmented set. Fig. 6, shows tractography plots obtained using Paraview for the caprine specimen in Fig.2, showing the patella tendon, anterior cruciate ligament (ACL) and the meniscus.

### 3.6 Reconstruction from experimental images with noise

The MR images obtained in the experiments were used to assess the performance of the method in the presence of image noise and the results are summarised in Fig.9 for different SNR values. In each case the SNR values were calculated as SNR=0.655∙S/σ, where S is the mean signal intensity and σ is the standard deviation of noise. S was taken to be the mean pixel intensity value of the segmented angle sensitive voxel in all 9 scans. The value of σ was found from the pixel intensities in a selected background region corresponding to background air. The factor 0.655 is due to the Rician distribution of the background noise in a magnitude image. This arises because the intensity variations due to added noise were all made positive, which artificially reduces.

Fig.7 summarises the results obtained for different SNR values. It presents sample images, including the original scans with SNR = 56, and those with increasing added noise, down to SNR = 5. Focussing on the patella tendon as the ROI, Fig. 7 also shows in each case the tractography plots, the fibre orientation distribution plots. In addition, Table 2 summarises the values of the calculated *Alignment Index* obtained for different SNR values. In all cases the direction corresponding to the peak of the orientation distribution was found to be (0.09, 0.12, 0.99), for which the maximum AI was calculated. The consistency of these results indicates that the method performs well even for images where the noise is quite significant. This is evident from the visual quality of the tractography plots, and also from the consistently similar number of segmented voxels, AI distribution plots and the maximum AI value in each case.

**Table 2:** Maximum alignment Index values calculated for different signal-to-noise ratios. In all cases the peak fibre direction was found to be (0.03, 0.23, 0.94).

Signal-to-Noise Ratio	56	30	20		10	5
Maximum Alignment Index	0.562	0.586	0.600		0.621	0.624

**Fig. 8:**
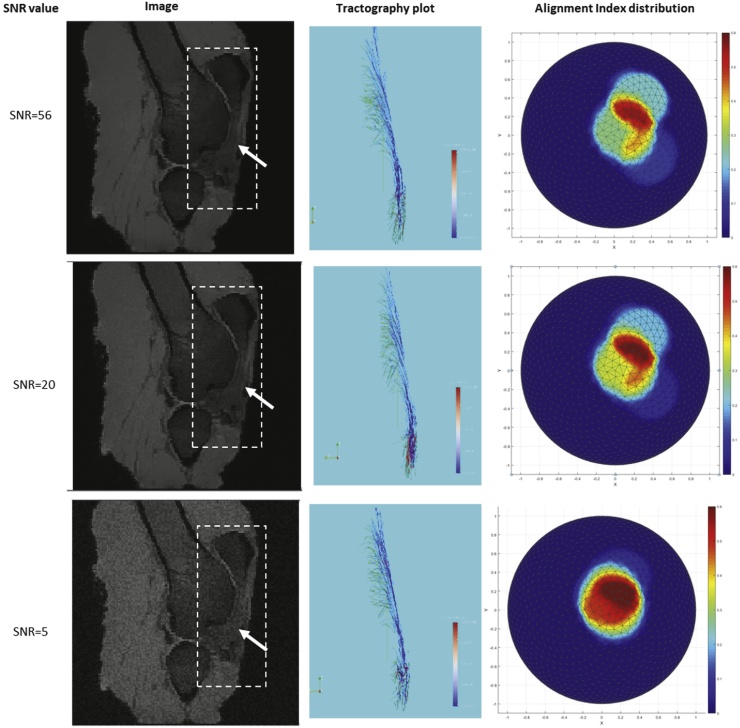
Percentage of failed fibre direction computations (error>10°) as a function of image SNR for the experimental images and Monte Carlo simulations. For the experimental images the original data with SNR = 56 was used as the gold standard, while the other images where those with synthetic noise added.

The robustness of calculating fibre orientations for different SNR values was measured by recording the percentage of failed calculations. Here, the original acquired images with no added noise (SNR = 56) were taken as the gold standard, yielding calculated fibre directions that were taken as absolutely accurate, and the results obtained from images with added noise were compared against those. A fibre estimation calculation was considered to fail if the resulting error was found to be >10°, consistent with the equivalent simulation studies in II.A. The results are presented in Fig. 8, which also includes the simulation results for comparison. The difference between the two plots can be attributed to a number of effects that are not accounted for in the simulations, including partial volume effects, the fact that voxels in different scan involve different orientations, non-uniformity of the actual tissue microstructure in ROI, as well as any errors due to registration.

## 4 Discussion

Methods exploiting the magic angle effect have the potential to provide valuable new diagnostic information about the tissue microstructures and can lead to reliable non-invasive diagnostic methods. However, it is difficult to envisage how such methods can be employed using conventional MRI scanners which use cylindrical magnets. The severely limited magnet confines make it impossible to change the angle between the main field and the subject, with few exceptions such as imaging of the wrist or the ankle. The situation is somewhat easier with open MRI in which the field is vertical and the patient is positioned between two magnet poles, although rotating the patient about two axis remains difficult to perform in practice. This issue can be solved by adopting novel MRI configurations involving a moveable magnet. Our group is currently developing a dedicated low-field scanner based on permanent magnets (McGinley et al., 2018), in which the magnetic field is parallel to the poles (transverse) and the magnet can be rotated about two axes (‘roll’ and ‘yaw’) while the patient remains stationary. In this way almost any desired field orientation can be achieved.

Methods such as Diffusion Tensor Imaging (DTI) have been employed to obtain collagen tractography plots (Van Dyck et al., 2017). In principle DTI does not require changing of the field angle as the diffusion tensor can be constructed by applying gradients in various directions. However, in order to obtain a sufficient MRI signal it is still found necessary to orient the anatomy at the magic angle, so again, these techniques cannot be readily applied in conventional scanners.

The conducted simulation studies conducted in this work were relatively simple, as they were intended only to provide a performance benchmark, with the noise as the main influencing parameter, and they were not intended to be a faithful simulation of MRI scanning at low field. For the purposes of the simulations, the values of parameters A and B (Eq. 3) were chosen according to the image intensity variation observed in experimental images. Those values can be expected to be different for a low field scanner and for different imaging sequences, and the value of B in particular will depend on the choice of TE. The values of A and B could be readily chosen for each image set according to the observed image intensity variation, as we did in this work. However, it is worth noting that our experiments with different values indicate that the performance of the fibre estimation method is quite insensitive to the choice of the parameter A. This could be explained by the fact that the method involves correlation, so it is the shape of the underlying curve of Eq. 3 that has the dominant influence, not the absolute values.

As mentioned above, the synthetic added noise in this work was Gaussian, involving only positive values in the range 0-255 which were added to the voxel intensity values. Strictly speaking, MR noise is known to have the Rician characteristic, but for noise values SNR≥3 this is known to be represented accurately by the Gaussian characteristic (Gudbjartsson and Patz, 1995). Therefore using Gaussian noise simplified the coding of the simulation without a significant sacrifice in accuracy. We accounted for the Rician nature of the background noise in the calculation of the resulting SNR, with the factor 0.655  in the formula SNR=0.655 S/σ.

We have shown that the number of scanning orientations can be reduced by as much as 50 % or more with an appropriate choice of directions, which adequately detect the angle-dependent variation in signal intensity. Simulation studies suggest that 9 equispaced scanning directions are sufficient to capture the angle sensitive signal intensity variation. However, even 7 equispaced scanning orientations may be sufficient for reasonable SNR. It should be noted that no a priori knowledge has been assumed about the expected orientation of collagen fibres, and we expect that in certain cases it would be possible to utilise such knowledge in order to reduce the required number of scans even further.

The proposed simplex minimisation method for estimating fibre orientations has been shown to achieve significant improvements over previous methods in both accuracy and speed, without the need to trade one against the other. The analysis of experimental data indicated that improved accuracy is particularly important for visualisation of orientation distribution plots, where lower accuracy can lead to less well defined distribution peaks and therefore less conclusive results. The main factors influencing accuracy are the number of scans and the SNR value.

Importantly, the method was shown to be robust and accurate in the presence of significant imaging noise. Reliable results were obtained even for quite low SNR values of about 6, and very consistent results were obtained for SNR > 10. It is reasonable to expect that much higher SNR can be routinely achieved in practice even with a low field system. This is a useful practical consideration as SNR may be traded to increase the overall scanning speed.

The adoption of the *Alignment Index* as a measure of 3D anisotropy, and visualisation using the AI plots, were found to be useful techniques for quantitative analysis of the results. The choice of 20° solid angle in this work was made as a compromise following different trial values, as we found that adopting a solid angle >10° led to consistent AI values across the range of SNR considered, while choosing an excessively large angle could compromise the usefulness of this metric.

A limitation of this study was that the accuracy of determining fibre orientations was not assessed against methods such as polarised light microscopy, which could provide a direct measurement of fibre orientations, as this equipment was not available. However it would be difficult to obtain results comparable to those presented here using such techniques, because they can deal with only a small tissue sample at a time and cannot assess the whole joint. Seidel et al. (Seidel et al., 2013) reported such comparison involving small tissue samples and a method similar to that by Szeverenyi (Szeverenyi and Bydder, 2011) with encouraging results. Our simulation studies did include the method in (Szeverenyi and Bydder, 2011) for comparison, and we were able to replicate their reported accuracy while demonstrating the improvements using the proposed simplex method.

It would also be useful to compare the tractography results with those obtained using diffusion tensor imaging (DTI) (Ferizi et al., 2017; Van Dyck et al., 2017), but this was not possible in the scope of our work. DTI typically requires long TE of 50−90 ms, which makes it difficult to obtain sufficient signal for tissues with short $T_{2}$ such as ligaments. This necessitates the use of suitably modified imaging sequences (Wengler et al., 2018) that provide a shorter TE, while it is also necessary to extend $T_{2}$ as much as possible by orienting the sample at the magic angle.

The current implementation of the method uses rigid body registration based on passive spherical markers, which was chosen for its accuracy, speed and convenience. Nevertheless, geometric distortions of images obtained at different orientations to the magnet, caused by the gradient non-uniformity and field inhomogeneity, pose a practical limitation. In the future we shall explore the use of soft registration methods (Klein et al., 2010) in this context, as well as the use of methods for accurate scanner calibration.

Our current research is directed towards providing high quality receiver coils for the prototype scanner such that consistent images are obtained at various arbitrary orientations of the subject and the receiver to the magnet. This will be followed by in-vivo studies using the methods presented.

## 5 Conclusions

The key achievement presented in this paper is the minimisation of the number of scans and a new method to compute fibre orientations which yields significantly improved accuracy and reduced computing times. The main factors influencing accuracy are number of scans and signal to noise ratio. Importantly, the method was shown to perform well in the presence of noise for quite low SNR values, and this is highly encouraging when considering the adoption of this approach for use with a low field MRI scanner. Plots of orientation distribution were found to provide a useful and intuitive method for results visualisation that can be used in combination with 3D tractography plots. The proposed alignment index was found to be useful metric for quantifying fibre anisotropy.

## CRediT authorship contribution statement

**Djordje Brujic:** Investigation, Formal analysis, Methodology, Software, Validation, Writing - review & editing. **Karyn E. Chappell:** Investigation, Validation, Writing - review & editing. **Mihailo Ristic:** Conceptualization, Funding acquisition, Methodology, Project administration, Supervision, Writing - original draft.

## Declaration of Competing Interest

The authors declare that they have no known competing financial interests or personal relationships that could have appeared to influence the work reported in this paper.
